# Screening and Biosensor-Based Approaches for Lung Cancer Detection

**DOI:** 10.3390/s17102420

**Published:** 2017-10-23

**Authors:** Lulu Wang

**Affiliations:** 1 School of Instrument Science and Opto-electronics Engineering, Hefei University of Technology, Hefei 230009, China; luluwang2015@hfut.edu.cn; 2 Institute of Biomedical Technologies, Auckland University of Technology, Auckland 1142, New Zealand

**Keywords:** medical imaging, magnetic induction tomography, lung cancer, biomarker, biosensor

## Abstract

Early diagnosis of lung cancer helps to reduce the cancer death rate significantly. Over the years, investigators worldwide have extensively investigated many screening modalities for lung cancer detection, including computerized tomography, chest X-ray, positron emission tomography, sputum cytology, magnetic resonance imaging and biopsy. However, these techniques are not suitable for patients with other pathologies. Developing a rapid and sensitive technique for early diagnosis of lung cancer is urgently needed. Biosensor-based techniques have been recently recommended as a rapid and cost-effective tool for early diagnosis of lung tumor markers. This paper reviews the recent development in screening and biosensor-based techniques for early lung cancer detection.

## 1. Introduction

Lung cancer is a major health problem in the United State and worldwide [1]. Every year, approximately 1.3 million new lung cancer cases and about 1.2 million lung cancer deaths occur in Europe and North America [2,3,4]. Previous studies have indicated that tobacco smoke [5], environmental pollution [6], second-hand smoke [7], industrial substances [8], and genetic factors [9] may cause lung cancer. Compared to some other common cancers such as breast cancer, lung cancer continues to have a much lower survival rate [10]. Early diagnostic of lung cancer with suitable treatment significantly improves the five-year survival rate [11]. Chemotherapy and radiation therapies are commonly applied for small cell lung carcinoma (SCLS) [12], while surgical treatments are normally provided for non-small cell lung carcinoma (NSCLS) [13]. Eastern Europe has the highest lung cancer mortality rate in males, while Northern Europe and America have the highest mortality rate in females [14]. Increases in new lung cancer cases are expected in some developing countries such as China and India in the next few years [15].

Researchers have extensively studied lung screening methods, including chest radiograph (CRG), computed tomography (CT), low-dose CT (LDCT), magnetic resonance imaging (MRI), and positron emission tomography (PET). These techniques have some drawbacks, such as being expensive and having low sensitivity for identifying cancer cells at early stages. Annual CRG was reported as not helpful in reducing the mortality of lung cancer [16]. CT has been considered as the gold standard lung screening tool, which offers information of tumor features such as size, characterization and tumor growth. 3D CT image offered assessment of the chest wall, diaphragm, and mediastinum invasion, in addition to staging of the tumor. Radiations produced from CT also increased the cancer risk [17]. To solve this limitation, LDCT was applied for lung imaging and it reduced 20% of lung cancer mortality [18]. However, LDCT continues to have a high false positive rate (up to 96.4%) [19]. 18F-Fluorodeoxyglucose PET/CT was applied in oncological imaging but produced inaccurate results [20,21]. Magnetic induction tomography (MIT) has been recently proposed for early disease detection with advantages of low-cost and high-sensitivity [22].

Apart from imaging approaches, biopsy is another common way to identify lung cancer; however, it is expensive and requires trained physicians [23]. Autoantibodies can detect lung cancer cells about five years earlier than autoradiography because tumor growth is associated with gene and protein changes [24]. Biomarker-based techniques for early diagnosis of tumor markers have attracted much attention [25]. The major limitations of these techniques include being time-consuming and expensive, and having low-sensitivity for low marker concentrations [26]. Therefore, it is necessary to develop a rapid, low-cost and high-sensitive method for early diagnosis of lung cancer. In recent years, various biosensors have been developed to analyze tumor markers for early diagnosis of various diseases including lung cancer [27,28,29,30].

This paper summarizes the recent achievements in screening and biosensor-based approaches for lung cancer detection. Several MIT sensor systems, including their benefits and limitations as well as future research directions are also reviewed. The paper is structured as follows: Section 2 presents the current available lung screening approaches; Section 3 describes the MIT based approaches for imaging of biological objects; Section 4 reviews some recently developed biomarker and biosensor based techniques for lung cancer detection; Section 5 presents some current trends and future perspectives of lung cancer detection techniques; and Section 6 concludes this paper.

## 2. Clinical Lung Screening Modalities

Table 1 compares some most popular screening methods for imaging of lung. Abnormal chest imaging is the conventional diagnostic method for lung cancer detection, which can be done by CT, LDCT, MRI, PET and bone scans [31].

### 2.1. Computed Tomography (CT)

CRG is the conventional screening modality for diagnosis of lesion and pulmonary nodule in subjects with high risk of lung cancer. It employs X-rays to produce lung images, which are associated with low sensitivity, high radiation exposure dose, and poor image quality. CRG is not helpful in reducing the mortality of lung cancer. Several clinical trials were conducted to investigate CRG and CRG with sputum cytology in the 1970s. The obtained results showed that both approaches have poor sensitivity and are not helpful in reducing lung cancer mortality [32,33].

CT can detect features of nodules such as characterization, size and tumor growth. 4D CT significant impacts lung cancer management by allowing more precise targeting of the administered radiation [34]. The major drawbacks of CT are cost, unsafe radiation exposure, high false-positive rate, and cannot be performed on a routine basis. The radiations produced from CT also increase the risk of cancer in children because they are more sensitive to radiation-induced carcinogenesis [35]. Additionally, there was poor correlation relationship between CRG and CT [36].

LDCT produces much lower radiations than CT, which has potential to become an effective tool for early lung cancer detection in high risk individuals [37,38]. The iterative image approach was developed to reduce dose in LDCT [39]. Lung LDCT was associated with a reduced mortality in high-risk population [40], while annual LDCT increased lung tumor incidence [41]. In a previous report, studies showed that LDCT detects more early-stage lung nodules and cancer cells than CRG, but it is not helpful in reducing lung cancer mortality [42,43,44]. Up to 90% of new lung cancer cases were detected using LDCT [45]. The national comprehensive cancer network published guidelines of lung cancer screening, and recommended annual LDCT for high-risk ex-smokers between 55 and 74 years who quit smoking within the last 15 years [46].

### 2.2. Positron Emission Tomography (PET)

PET is an accurate tool for identifying lung nodules and detecting metastases from malignant tissues. Compared to CT, PET offers higher sensitivity and higher specificity for lung cancer detection [47,48]. A previous published report showed that PET has a high false-positive rate, especially with larger nodes (>1 cm) due to reactive or granulomatous nodal disease [49]. PET with F-18 deoxy glucose (FDG) was recommended to be a useful and cost-effective approach in the management of solitary pulmonary nodules. The sensitivity and specificity of PET-FDG are 96% and 80%, respectively, for lung tumor detection [50,51]. The false-positive rate of PET is highly related to the clinical context and the prevalence of granulomatous and infectious disease [52].

PET is a cost-effective tool for diagnosis of NSCLC [53]. FDG-PET and PET play an important role in patient selection and target volume definition in subjects with advanced NSCLC for radical radiotherapy. Radical radiotherapy was given with curative intent to non-surgical subjects with gross locoregional tumor that can be encompassed by high-dose radiation in the absence of distant disease [54]. It was reported that PET-assisted radiotherapy treatment is more accurate than the conventional radiotherapy treatment [55]. Approximately 32% of lung cancer patients at stage IIIA can be managed by the PET/CT-assisted radical radiotherapy [56]. PET offers superior correlation with longer time to progression and overall survival. FDG-PET offers prognostically significant response assessment in NSCLC subjects undergoing induction chemotherapy.

### 2.3. MRI

MRI is a powerful tool for lung imaging without ionizing radiation. However, it was not recommended for regular lung screening because it provides insufficient anatomic information, and is time-consuming and expensive. Published results showed that lung MRI detects up to 90% of nodules 4–8 mm in diameter and 100% of nodules >8 mm in diameter [57]. The respiratory-gated proton MRI was investigated for small mice neoplastic lesion detection [58]. Investigators also studied the optimized proton MRI sequences for imaging of lung [59].

MRI with ultra-short echo-time (UTE) pulse sequence is extremely efficient for limiting motion artifacts. MRI with UTE helps to enhance the MRI signal intensity of pulmonary tissue and reduces lung susceptibility artifacts. Researchers have investigated MRI with gradient echo and MRI with T_2_-weighted fast spin echo for diagnosis of lung nodules (3–4 mm in diameter) [60]. It was found that MRI with UTE is more sensitive than MRI with 3D dual-echo GRE for small pulmonary nodule detection (4–8 mm) [61]. However, further investigations are needed to improve the spatial resolution and robustness of lung MRI.

Lung MRI has a higher false-positive rate (up to 95%) than LDCT, and the detection rate and sensitivity of MRI are satisfactory. It is hard to detect lung nodule with MRI due to the low intensity of nodule. Fink et al. [62] investigated the sensitivity of MRI for lung nodule detection by using different pulse sequence. Cieszanowski et al. [63] applied MRI with T_1_-weighted sequence and MRI with T_2_-weighted sequence for diagnosis of small lung nodules, respectively. Their research findings showed that the false-positive rate is affected by types of sequence. MRI comprising two or three sequences is helpful in reducing the false-positive rate. It was found that 3T MRI has more difficulty detecting ground glass opacities than 1.5T MRI [64]. 1.5T MRI with SSFP sequences successfully detected ground glass opacities in 75% of lung fibrosis subjects [65]. MRI with T_2_-weighted fast spin echo for ground glass infiltrates detection was similar or even better in immunocompromised subjects [66].

### 2.4. Breath Test

Breath analysis or breath test is one of the most noninvasive screening approaches for early diagnosis of lung cancer, which has gained attention due to its easy and fast data collection process [67,68]. Breath gas mainly contains nitrogen and carbon dioxide produced by respiration. It consists of more than 100 gas species with different concentrations, which may provide useful information for early diagnosis of disease [69]. However, breath test has some drawbacks, such as insufficient accuracy because exhaled air has many crucial volatile organic compounds (VOCs) at very low concentrations and there is no clear protocol for breath sampling [70]. To solve these challenges, many researchers have investigated gas chromatography–mass spectrometry (GC/MS), however, this method is expensive and it is not easy to build an implementation system.

VOC patterns (multiple VOCs) have been reported as useful biomarkers for exhaled breath analysis for disease detection [71,72,73]. Various algorithms have been developed to measure VOC patterns for lung cancer detection, including forward stepwise discriminant analysis [74], support vector machine (SVM) [75], weighted digital sum discriminator [76], logistic regression [77], partial least-squares regression [78] and random forest classification [79].

Recently, Sakumura et al. [80] developed a prototype system to measure exhaled breath made of simple GC columns, a simple gas-condenser unit and SnO_2_-based semiconductor gas sensors. To find the most effective combination of VOCs for lung cancer detection, they analyzed the VOCs measurements from lung cancer patients and healthy subjects. They also performed a subsequent statistical analysis to detect lung cancer by combining various VOCs. Their research findings indicated that the five-element VOC pattern of CHN, methanol, CH3CN, isoprene, and 1-propanol is sufficient for 89% screening accuracy [69].

## 3. Magnetic Induction Tomography and Measurement Systems

**Figure 1:**
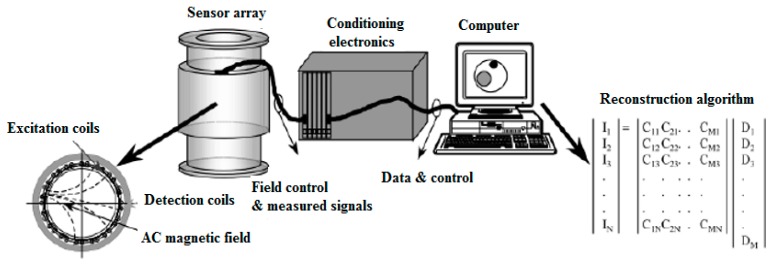
Diagram of magnetic induction tomography system [85].

Recently, MIT has been proposed as a new cost-effective imaging tool for diagnosis of various diseases including lung cancer and brain stroke [81,82,83]. Similar to electrical impedance tomography (EIT), MIT measures the electromagnetic properties of biological object. Compared to EIT, MIT can obtain more information on insulating tissue such as ribcage [84]. As shown in Figure 1, a MIT system normally contains several coils (excitation coils and detection coils) arranged around the object to generate and measure the scattered magnetic field, and a host computer with matched software to produce images [85]. During data collection, an excitation coil generates primary field B_0_ in the conductive medium, the induction of eddy currents accompanies the interaction in the medium itself as the primary field propagates and penetrates the medium. The secondary field ∆B is also known as the magnetic perturbation field [86].

Al-Zeibak et al. [87] applied the MIT theory to distinguish fat tissue and water-bearing fat free tissue. Most MIT hardware systems are dependent on external devices and power amplifiers to produce high-power signals with less noise. MIT-based techniques have not been extensively investigated in clinical environments due to the limited image resolution, and they have not met the standard for widespread commercialization. To solve these problems, investigators have developed several approaches with particular focus on the development of clinical applicable MIT systems.

### 3.1. Gradiometer

Gradiometer is attractive for magnetic field measurements in noisy environments, which is commonly applied in MIT systems to measure the gradient of magnetic field. Axial gradiometer, planar gradiometer (PGRAD) and asymmetrical gradiometer are the three main types of gradiometers. The axial gradiometer has been employed in various MIT systems to remove the primary field. Karbeyaz et al. [88] applied a single coaxial gradiometer to move over the phantom using the MIT system. Riedel et al. [89] investigated the precision and sensitivity of an axial gradiometer containing five PCBs covered with shielding layers to avoid capacitive coupling.

Xu et al. [90] developed a multi-channel hemispherical system with several modifications to the circuit of Riedel’s system. They improved the stability of the Riedel’s system by using a cancellation senor to reduce the phase drift. They also used an amplifier with high CMRR for capacitive coupling rejection, whereas shielded cables were applied to remove unwanted signals. Various PGPADs have been developed for MIT applications; the thin-film PGRAD is more attractive to measure the magnetic field, and can be fabricated with high intrinsic balance owing to the precision photolithographic methods applied to fabricate these devices.

Ketchen et al. [91] first developed a thin-film PGRAD, which was made of parallel and series configured pickup loops coupled to the superconductivity quantum interference device (SQUID) inductance. Since then, various planar gradiometers with baseline less than 2 cm have been investigated. Stolz et al. [92] developed a PGRAD with two series-configured pickup loops transformer coupled to a thin-film SQUID. Cantor et al. [93] developed a similar long-baseline planar gradiometer.

Scharfetter et al. [94] developed a coil PGRAD system with the receiver coil and excitation coil located at the same position. The primary signal was subtracted from the captured signal (measured by reference coil). The signal to noise ratio was improved significantly by applying the planar gradiometer. Rosell et al. [95] evaluated the coil PGRAD system analytically and experimentally. PGRAD offered a robust and stable cancellation technique capable of reducing carrier signal in the absence of conductivity perturbations while maintaining essentially the same absolute sensitivity for local perturbations. PGRAD system required fewer electronic devices than the coil system. Compared to PGRAD system with 16 coils, the PGRAD system with 16 gradiometers has lower sensitivity.

Scharfetter et al. [96] investigated the PGRAD and solenoid coil using the single channel MIT system. PGRAD was less sensitive to far-field electromagnetic interference and produced phase errors due to the thermal mismatches between gradiometers halves. The same research team also developed a multi-channel MIT imaging system including zero flow gradiometer (ZFGRAD) that combines the benefits of PGRAD and zero flow coil (ZFC) [97]. ZFGRAD offered better immunity to far magnetic perturbation than PGRAD and ZFC (up to 12 times). ZFC and ZFGRAD exhibited their maximum sensitivities near the tank borders on the side of the excitation coil, whereas the PGRAD achieved more sensitivity near the receiver side.

Merwa et al. [98] developed a MIT system comprising 16 excitation coils and 32 receiving coils. The MIT system with 32 receiving coils offered good localization of perturbed sphere. The system with 16 PGRADs offered better localization; however, two places had ghost images with opposite sign mirrored true image with respect to the x-y plane. The point spread function was applied to solve the “location unrecognized” problem. Such function defined the propagation of image due to a point source or object as related to the location and geometry of the perturbation [99].

### 3.2. Excitation Coil

Coil sensor plays an important role in MIT systems, which is sensitive to the flux. Many sensors have been investigated for implementing MIT systems, such as Rogowski coils, gradiometers, vibrating coils, tangential field sensors and needle probes [100]. Air-core coil and ferromagnetic core coil are the most commonly available excitation coils, and helpful in reducing the effect of the primary field. The ferromagnetic core coils can overcome the low sensitivity of air-core coil and act as flux concentrator.

Various types of receiving coils have been developed to reduce the effects of primary field, such as excitation and receiver coil with back-off coil, planar array, axial gradiometer, PGRAD-differential coil, ZFC-receiver coils, and ZFPGRAD-planar gradiometer [101,102]. Stawicki et al. [103] developed an excitation coil that contains a conducting shield to protect the primary field. The ferrite core was located at the center of screen and it could concentrate the primary field in the core material itself; such design increased the magnetic field. Barba et al. [104] used a similar measurement system to conduct their investigations.

### 3.3. Sensor Arrangement

The image quality of MIT system is highly dependent on the sensor array configurations. Watson et al. [105] recommended that the primary field from the planar array can be done with a sensor coil. Igney et al. [106] enhanced Watson’s work by using a shielded PCB printed coil. Such design improved the efficiency of the insensitivity to the primary field effects. Eichardt et al. [107] investigated the performance of MIT systems by using different sensor arrays including cylindrical and hemispherical sensor arrays. Compared to the cylindrical system, the hemispherical MIT system offered higher sensitivity, and larger coil sensors were more sensitive in relation to standard setups. The sensitivity was increased by increasing the distance between each two-coil element.

Gursoy et al. [108] studied six sensor arrays and they found that the sensor arrays affect stability and image quality significantly. They also developed a fast-deterministic algorithm to optimize the sensor arrays, which has the ability to produce the most suitable sensor array, even though it does not guarantee finding the global optimum. Scharfetter et al. [109] proposed an active marker system for artifact suppressions during object movements. The system contained an elastic belt with several small loops that controlled by the data collection unit. More accurate images were obtained when the assumed SNR close to the determined. Results showed that MIT is possible for tracking of object boundaries. However, future investigations are needed with particular focus on the development of biomarkers and the optimization of working frequencies.

### 3.4. Recent Development of MIT

MIT has recently been applied for medical applications with particular focus on imaging of lung, brain, heart, liver tissue and biological tissues [110,111,112,113,114]. Gabriel et al. [115,116] investigated various biological tissues over a wide frequency range. The simplicity of characterizing passive electrical properties of biological tissues offered an alternative way for imaging of biological objects. Table 2 compares various newly developed MIT systems. Patz et al. [117] developed a MIT with direct digitizing signal measurement (DDSM) module to detect cerebral stroke. The National Instrument (NI) PXI system was applied to conduct the experimental work with working frequency of 10 MHz. The amplifier was applied to improve the measurement phase delay.

**Figure 2:**
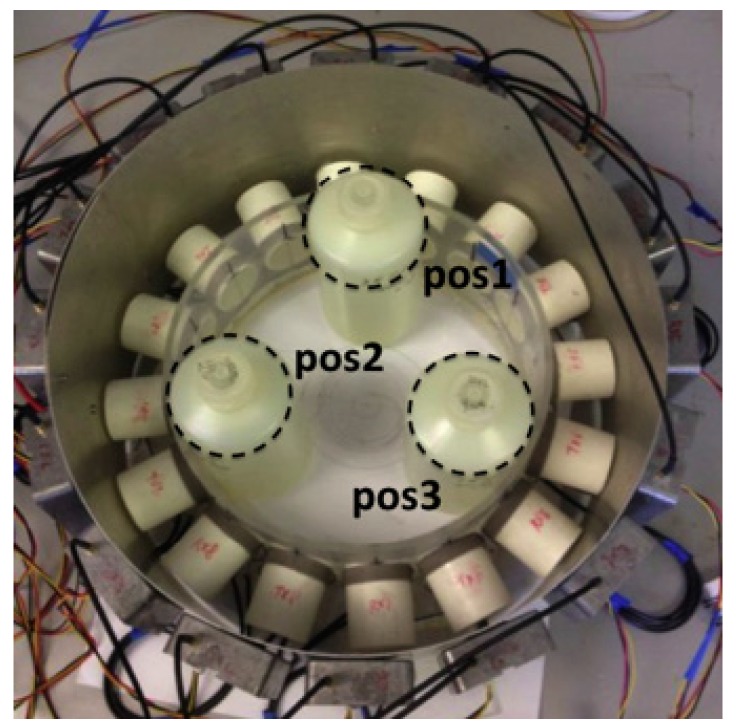
Photo of the 16-channel MIT measurement setup for saline bottle detection [84].

Referring to Figure 2, researchers at the University of Bath have developed a 16-channel MIT system for biomedical applications [84]. In their experimental setups, the NI system was applied to accomplish the signal driving, switching and data acquisition; such design simplified the system while providing satisfactory performance. The system was experimentally validated to detect a saline bottle. Results showed that the MIT system has potential for biomedical imaging applications [118].

Watson et al. [105] developed a MIT with phase-stable amplifier for biomedical application. The phase-stable amplifiers and the gradiometers configurations need to be mutually exclusive, and the highest measurement precision could be achieved by utilizing both approaches. The MIT image quality would be improved by increasing the number of coils, however, such method also increased the system cost, complexity and operation time. A rotational MIT system containing a transceiver RF coil was developed for biomedical application. Compared to conventional systems, the proposed rotational system offered a better field penetration depth towards the center of image. The exiting MIT systems suffer from several limitations. The capacitive coupling between excitation and receiving coils affects the measured data from receivers; therefore, it is important to eliminate capacitive coupling to represent the actual results [119].

**Figure 3:**
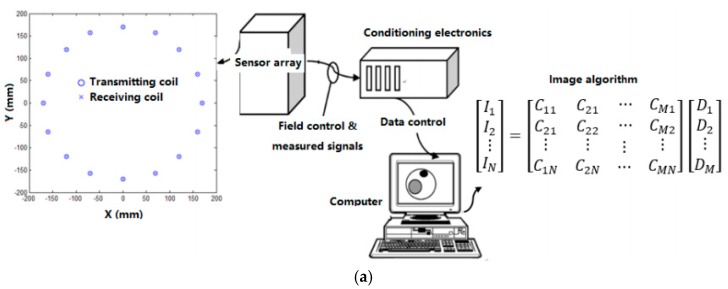

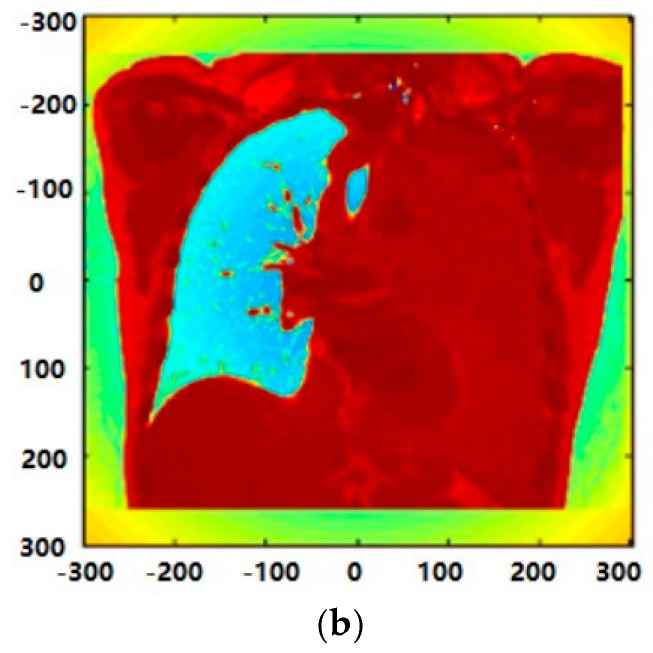
(**a**) Diagram of holographic electromagnetic induction (HEI) system; and (**b**) simulation result of lung phantom using the HEI system [120].

More recently, Wang et al. [120] developed a numerical model of holographic electromagnetic induction (HEI) system for biomedical applications with particular focus on lung cancer detection based on MIT technique. The system (see Figure 3) is made of 16 coils, and each coil worked as transmitter and receiver. Various simulations were conducted with several realistic human thorax models and results demonstrated that HEI could detect arbitrary shaped lung tumors with random sizes and locations. The research outcomes offered crucial priority information that can be exploited to improve MIT based approaches.

## 4. Biomarkers for Lung Cancer Detection

Biomarkers predict the response to certain types of therapy such as surgery and chemotherapy or estimate the risk of future relapse. Genetic and proteomics-based biomarkers are two major types of biomarkers, which can be identified through tumor cells, urine, sputum, blood, or other body fluids [3]. Table 3 demonstrates numerous lung cancer markers [121,122,123,124,125,126,127,128,129,130,131,132,133,134,135,136,137,138,139,140,141].

### 4.1. Proteomic Biomarkers

Many types of proteomic biomarkers have been investigated for lung cancer detection, including Annexin II [122], APOA1 [123], carcinoembryonic antigen (CEA) [124], carbohydrate antigen 125 (CA125) [125], carbohydrate antigen 19-9 (CA19-9) [126], cytokeratin fragment 21-1 (CYFRA21-1) [127], CD59 glycoprotein [128], transthyretin (TTR) [129], GM2 activator protein (GM2AP) [130], haptoglobin-R2 [131], Ig-free light chain [132], neuron-specific enolase (NSE) [133], nitrated ceruloplasmin [134], plasma kallikrein B1 [135], ProGRP [136], retinol binding protein (RBP) [137], squamous cell carcinoma (SCC) [138], vascular endothelial growth factor (VEGF) [139], TPA [140], and tumor M2-pyruvate kinase [141].

CEA is a common proteomic biomarker to distinguish malignant tissue and benign tissue. The CEA level range of 2.5–5 ng/mL was observed in healthy subjects, and the highest level of CEA offered a useful prognostic indicator [142]. NSE was applied as the putative serum marker of SCLC with relatively higher sensitivity and specificity compared to CEA [143,144]. A subject is suspected suffering from SCLC if the NSE level is greater than 35 ng/mL. Annexin II and ENO1 are other common lung cancer biomarkers [145]. Up to date, it is still not possible to detect lung cancer with a specific biomarker, as most identified markers are nonspecific indicators. Therefore, a protein biomarker panel was applied for the accurate detecting of disease consisting of CEA, retinol binding protein (RBP), R1-antitrypsin (AAT), and squamous cell carcinoma (SCC) antigen. The proteomics-based biomarker panel detected about 88% of subjects with lung cancer and 82% of subjects without cancer [146]. The sensitivity was improved with a biomarker panel containing CEA and some specific biomarkers such as ENO1, SCC, NSE, and CYFRA21-1 [147].

CYFRA21-1 protein has been recognized as the most sensitive biomarker to identify NSCLC, especially squamous cell carcinoma [148]. Previous research findings have confirmed the sensitivity and specificity of CYFRA21-1 as a tumor marker [149]. CYFRA21-1 gene has been reported as the most robust DNA-based biomarker for NSCLC [150]. Subjects with advanced NSCLC observed high-level serum CYFRA21-1 [151]. NSCLC patients with high-level serum CYFRA21-1 are poor prognosis. High serum CYFRA21-1 level may be a useful noninvasiveness marker to identify NSCLC risk, however, this statement requires further clinical investigations.

### 4.2. Gene Biomarkers

This section reviews the most commonly available genetic lung cancer biomarkers, including p53, p16, K-ras, telomere length and telomere-related genes and microRNAs. p53 mutation occurred in 50% of subjects with NSCLCs and the spectrum changes around 34–82%. p53 expression occurred in about 58% of subjects with lung cancer [152]. Additionally, there was a correlation between bcl-2 and p53 over-expression for lung cancer patients [152].

p16 plays a crucial role in regulating cell cycle. p16 methylation normally occurs in lung cancer patients especially chromate lung cancer and smokers. Approximately 21–51% of NSCLC patients observed p16 methylation, and about 54–100% of NSCLC patients observed p16 loss of heterozygosity [153,154]. p16 gene is normally associated with lung cancer, which can be affected by tobacco smoking, and radon and plutonium exposure. It was reported that p16 methylation is highly related to past smoking and duration of smoking [155].

Ras genes are responsible for cancer-causing activities of Harvey (H-ras) and Kirsten (K-ras) sarcoma viruses. Ras mutation was observed in about 20–25% of cancer patients and up to 90% of patients with specific cancers [156]. K-ras, H-ras and N-ras code 21 kD proteins are the three main types of human Ras genes; they are members of Ras superfamily of GTPases and have a crucial role in cell proliferation and signal transduction. Approximately 60% of Ras mutations are confined to codon 12 of K-ras [157]. Approximately 78% of lung cancer patients observed K-Ras mutation, and subjects with NSCLS, pleural effusion, sputum, serum and bronchoalveolar lavage fluid also observed K-Ras mutation [158].

Lung diseases are also highly related to telomere-related genes such as TERT, TERF2 and POT1 [159,160]. The shortening in telomere length is related to cancers and the genetic alterations in telomere-related genes change the risk level of cancer due to telomere length. The risk of lung cancer is increased with the longer tertile of telomere.

The serum miRNAs have potential to detect some diseases include lung cancer. Comprehensive studies of miRNAs have been performed for diagnosis of lung cancer [161]. Several miRNAs have been investigated extensively for target lung marker detection [162]. Additionally, serum miR-206 and miR-133b were applied to detect lung carcinogenesis [163]. High hsa-mir-155 and low hsa-let-7a-2 were used to identify lung tumor markers [164]. MiR-449c with the direct target of c-Myc has been investigated to identify NSCLS, which could suppress cancer cells growth in vivo [165]. It was found that tumor growth is associated with silence and overexpression of miRNAs, and overexpression of miRNAs is suitable for early lung disease detection.

### 4.3. Biosensors for Lung Cancer Biomarker Detection

Enzyme-linked immunosorbent assay (ELISA) is the conventional approach for biomarker detection. It requires a labeling process, which hinders monitoring of the probe/target interaction rapidly. To overcome this challenge, investigators have studied many high-sensitive and label-free detection techniques such as Field effect transistors (FETs), surface plasmon resonance (SPR) and quartz crystal microbalance (QCM). Among these techniques, FETs are more attractive because they are compact, inexpensive and able to integrate many sensors on the same chip. Over the years, researchers worldwide have paid attention to developing label-free and high-sensitive biosensors for early diagnosis of diseases such as lung cancer. Table 4 lists some recently developed biosensors for lung tumor marker detection [166,167,168,169,170,171,172,173,174,175,176,177,178,179,180].

#### 4.3.1. Optical Biosensors

Various optical-based biosensors have been developed for early diagnosis of lung cancer markers and the techniques have been improved by applying nano-techniques and surface chemistry [170,171,172,173,174,175,176,177,178,179,180]. Existing optical biosensors can be divided into six main groups: fluorescence, interferometric, SPR, optrode-based fiber, evanescent wave fiber, and resonant mirror optical biosensors. Currently, most commercial platforms use fluorescence detection systems, while most research tools use grating coupler and resonant mirror systems.

SPR-based biosensors have been developed for biomolecular interaction [173,174,175,176,177,178,179,180,181,182,183,184,185]. SPR-based sensors excite surface plasmon from the interface and measure the refractive index changes, which can be classified as label-free and real-time affinity reaction detection systems. A collimated polychromatic light beam from a halogen lamp passes through an optical prism and contacts a thin gold layer at a defined angle of incidence. Upon the incidence of the thin gold film, each light beam excites a surface plasmon at a certain wavelength. The reflected light is collected by an instrument with measured channels. Various SPR and FET based biosensors have been developed for assaying CYFRA21-1 protein [182,183].

Wang et al. [186] developed a high-precision optical system based on magnetic ELIA to detect CYFRA21-1. The system has potential to become a powerful tool for the rapid detection of lung cancer marker with advantages of compactness and high-sensitivity. Recently, Ribaut et al. [187] developed an innovative plasmonic optical fiber immunosensor to detect lung cancer marker cytokeratin 17. The proposed optical fiber immunosensor was tested on human lung biopsy, and the accurate detection of biomarkers in soft matters including tissues could be performed with plasmonic optical fiber grating immunosensors. Their research outcomes offered significant contributions toward diagnosis of biomarkers in tissues in clinical environments.

#### 4.3.2. Piezoelectric Biosensors

Piezoelectric biosensors are helpful for bioanalytical applications with several advantages such as easy-to-make, cost-effective and high-sensitivity. Piezoelectric quartz crystal (PQC), a thin slice of quartz derived from a single crystal with optimal chemical, electrical and mechanical properties, is suitable for analytical applications. A vapor deposition of gold or silver was fabricated on a PQC sensor to serve as electrodes.

QCM-based sensors have been applied for point mutation detection of lung cancer [188,189,190,191,192,193]. They measure frequency changes in quartz crystal resonators based on adsorbate recognition, and mass changes caused by selective binding can be detected by the corresponding changes in crystals. Quartz is the most popular crystal for analytical applications due to the mechanical, electrical and chemical properties of quartz, which demonstrate piezoelectricity effectively. QCM has the ability to diagnosis small objects, such as molecular weight ligands, cells, viruses, proteins and nucleic acids [191]. Piezoelectric immunosensors with specific antibodies have been applied to detect cancer makers. Further, minimal mass threshold for unit change of frequency in conventional QCM has been improved using nanoparticles, which led to ultrasensitive detection of analytes up to few attomoles.

#### 4.3.3. Electrochemical Biosensors

In recent years, various electrochemical biosensors have been developed for lung cancer marker detection [194,195,196,197]. Electrochemical-based sensors normally contain semiconductors and screen-printed electrodes, which can detect various molecules including proteins, antibody, DNA, antigen and heavy metal ions. Electrochemical biosensors are highly sensitive for diagnosis of lung cancer markers. Recent advances in electrochemical nano-biosensors offer a promising tool for diagnosis of molecules with some advantages, including low-cost, more accurate, fast-response and high-sensitivity.

Altintas et al. [195] developed a magnetic particle-modified capacitive sensor to detect some cancer markers such as CEA, CA15-3 and hEGFR. Experimental validations have been conducted to evaluate the proposed sensor. CEA and hEGFR were detected in the concentration range of 5 pg/mL to 1 ng/mL while CA15-3 was detected in the range of 1–200 U/mL with high specificity. The experimental results showed that the proposed sensor may become a useful tool for early cancer detection.

Recently, Tabrizi et al. [196] developed a highly sensitive electrochemical aptasensor based on carbon–gold nano-composite modified screen-printed electrode. VEGF165 was observed in lung cancer patients by using the proposed sensor, which might have potential to become a powerful tool for lung cancer detection. More recently, a new aptamer-based electrochemical sensor was developed by Zamay et al. [197] to identify lung cancer. The sensorcould identify cancer-related targets in crude blood plasma of lung cancer patients.

## 5. Current Trends and Future Perspectives

The currently available lung screening approaches are effective but have some drawbacks, as detailed above. MIT technique has potential to become an additional or alternative method to CT for lung disease detection. MIT-based approaches have some challenges, including heavy computational imaging algorithm, unrealistic thorax phantoms, difficult hardware systems for clinical use, and spatial resolution. To overcome these challenges, it is necessary to develop a high dynamic hardware implementation system to detect the small difference of scattered electromagnetic field.

Many investigators have employed more MIT coils to increase image resolution. This method also increased the mutual coupling signals between coils, which may reduce the accuracy of detection. Moreover, the cost and complexities of the implementation systems are also increased using the method of increasing coils. To solve this problem, a single coil can be applied to replace the multi-coil array. Recent studies have suggested that optimization of coil array configurations offer some potential benefits such as high image resolution, low-cost, and reduced operating time. Multiple in multiple out technique may also helpful in reducing complexity of the hardware system. However, further MIT investigations should be taken to improve the image algorithm and the implementation system with particular focus on the development of low-cost and compact RF coils and coil arrays to improve the image quality.

Biosensor-based techniques with particular focus on diagnosis of tumor markers have gained worldwide attention in the past few years. Up to date, biosensor and biomarker based techniques are still immature and the obtained evidence is too restricted for early diagnosis of lung cancer. Proteomic biomarkers have been applied within a panel of protein biomarkers, but they were not recommended for diagnosis with individual biomarker. The individual marker was not helpful for clinicians to obtain sufficient information about cancer tissues such as cancer stage, treatment and patient information. The major drawback of biosensor-based techniques is related to integration of lung cancer detection in primary healthcare. Among all these biosensors, QCM biosensors are more suitable and reliable for clinical surgery. The limitations of biosensors also include small target size, marker levels, and the possibility of high non-specific binding in the case of serum or real patient samples. Nano-biosensor techniques for molecules detection offer great potential for early lung cancer detection, however, these techniques are immature for clinical trials. More investigations should be provided to improve the sensitivity, accuracy, and multiplexing capacity of biosensors in the future.

## 6. Conclusions

This manuscript reviewed the recently developed imaging and biosensor-based techniques for early diagnosis of lung cancer. Recent developments in screening techniques such as CT, PET, MRI and breath analysis, as well as MIT-based approaches, were addressed with particular focus on for early lung cancer detection. Clinical trials of MIT-based approaches for lung disease detection caused worldwide excitement, which confirmed that MIT has potential to become a low risk alternative or additional clinical tool to CT for lung disease detection. Some recently developed biosensor-based techniques for diagnosis of target lung tumor markers were also reviewed. However, MIT and biosensor-based approaches are still immature for clinical applications across large populations.

## Figures and Tables

**Table 1:** Conventional lung screening methods [31].

Type	Advantage	Disadvantages	Time
Chest X-ray	Reliable	Produces radiations, low sensitivity, low specificity	few seconds
CT	Reliable	Expensive, high false-positive rate, low sensitivity, produces radiations	5 min
MRI	Reliable	Expensive, unsuitable for all cancers	40–60 min
PET	Reliable	Expensive, radioactive substance and sophisticated instrument are required, unsuitable for patients with other complications	90–240 min

**Table 2:** The currently proposed MIT systems.

	Frequency	Sampling Rate	Driving Level	Phase Noise (m^o^)	Phase Drift (m^o^)	Linearity
Bath Medical system	10 MHz	100 MS/s	30 mA	4	25	R^2^ = 0.9996
Cardiff Mk2 system [106,117]	10 MHz	120 MS/s	100 mA rms	9	119	R^2^ = 0.9998
CrazMk2 system [118]	50 kHz–1.5 MHz	60 M/s	Max. 200 mA	N/A	N/A	N/A
Glamorgan system [113]	10 MHz	N/A	N/A	N/A	27	N/A
Phillips system [114]	10 MHz	192 kS/s	50 mA rms	12.5	102	R^2^ = 0.9878

**Table 3:** Lung cancer markers.

Type	Biomarker
Proteomic biomarkers	Annexin II [122], APOA1 [123], CEA [124], CA125 [125], CA19-9 [126], CYFRA21-1 [127], CD59 glycoprotein [128], TTR [129], GM2AP [130], haptoglobin-R2 [131], Ig-free light chain [132], NSE [133], nitrated ceruloplasmin [134], plasma kallikrein B1 [135], ProGRP [136], RBP [137], SCC [138], VEGF [139], TPA [141], tumor M2-pyruvate kinase [141], ENO1
Gene biomarkers	p53, p16, K-ras, microRNAs, miR-21, miR-210, miR-182, miR-31, miR-200b, miR-205, miR-183, miR-126-3p, miR-30a, miR-30d, miR-486-5p, miR-451a, miR-126-5p, miR-143, miR-145, miR-206, miR-133b, hsa-mir-155, hsa-let-7a-2, TERT, TERF2, POT1, MiR-449c

**Table 4:** Some new developed biosensors for target marker detection.

Biosensor	Biomarker	Capture Agent	Sample	Limit of Detection	Linear Range	Ref.
Electrochemical	VEGF	VEGFreceptor-1	Serum	~	10–70 pg/mL	[167]
		Aptamer	~	15 nM	~	[168]
	p53	ssDNA	~	~	~	[169]
Fluorescent	VEGF165	Aptamer	Serum	~	1.25 pM–1.25 μM	[170]
	COX-2	Polyclona antibody	Blood sample	1.02 × 10^−4^ ng/mL	7.46 × 10^−4^ –7.46 × 10 ng/mL	[171]
SPRi-MALDITOP MS	LAG3 protein	Antibody	Plasma	~	~	[172]
SPR	TP53 gene	DNA		~	0.3–2 μM	[173]
	CEA	Antibody	Serum	~	~	[174]
	p53	p53 antigen	Serum	~	20 ng/mL–20 μg/mL	[175]
	p53	ds-DNA & antibody	~	10.6 and 1.06 pM	~	[176]
	EGFR	Aptamer	Serum	~	~	[177]
	CA19-9	Antibody	~	66.7 U/mL	~	[178]
	DNA mutations	ssDNA	Serum	50 nM	~	[179]
	K-ras mutation	PNA	~	~	~	[180]

## References

[1] World Health Organization Cancer Fact Sheet 2017. http://www.who.int/mediacentre/factsheets/fs297/en/.

[2] Lin W.Y., Hsu W.H., Lin K.H., Wang S.J. (2012). Role of preoperative PET-CT in assessing mediastinal and hilar lymph node status in early stage lung cancer. J. Chin. Med. Assoc..

[3] Zhang Y., Yang D., Weng L., Wang L. (2013). Early lung cancer diagnosis by biosensors. Int. J. Mol. Sci..

[4] Hasan N., Kumar R., Kavuru M.S. (2014). Lung cancer screening beyond low-dose computed tomography: The role of novel biomarkers. Lung.

[5] Yuan J.M., Nelson H.H., Carmella S.G., Wang R., Kurigerlaber J., Jin A., Adams-Haduch J., Hecht S.S., Koh W.P., Murphy S.E. (2017). Cyp2a6 genetic polymorphisms and biomarkers of tobacco smoke constituents in relation to risk of lung cancer in the Singapore Chinese health study. Carcinogenesis.

[6] Besaratinia A., Pfeifer G.P. (2008). Second-hand smoke and human lung cancer. Lancet Oncol..

[7] Alexander B.H., Checkoway H., Wechsler L., Heyer N.J., Muhm J.M., O’Keeffe T.P. (2015). Lung cancer in chromate-exposed aerospace workers. J. Occup. Environ. Med..

[8] Sullivan I., Salazar J., Arqueros C., Andrés M., Sebio A., Majem M., Szafranska J., Martínez E., Páez D., López-Pousa A. (2017). Kras genetic variant as a prognostic factor for recurrence in resectable non-small cell lung cancer. Clin. Transl. Oncol..

[9] Lee H., Jin G.Y., Han Y.M., Chung G.H., Lee Y.C., Kwon K.S., Lynch D. (2012). Comparison of survival rate in primary non-small-cell lung cancer among elderly patients treated with radiofrequency ablation, surgery, or chemotherapy. Cardiovasc. Interv. Radiol..

[10] Reed M.F., Molloy M., Dalton E.L., Howington J.A. (2004). Survival after resection for lung cancer is the outcome that matters. Am. J. Surg..

[11] Chiang T.A., Chen P.H., Wu P.F., Wang T.N., Chang P.Y., Ko A.M., Huang M.S., Ko Y.C. (2008). Important prognostic factors for the long-term survival of lung cancer subjects in Taiwan. BMC Cancer.

[12] Hara N., Ichinose Y., Motohiro A., Noge S., Miyake J., Ohta M., Hata K. (1986). Combination chemotherapy and radiation therapy for small cell carcinoma of the lung. Gan Kagaku Ryoho Cancer Chemother..

[13] Stamatis G., Eberhard W., Pöttgen C. (2004). Surgery after multimodality treatment for non-small-cell lung cancer. Lung Cancer.

[14] Malvezzi M., Bertuccio P., Rosso T., Rota M., Levi F., La V.C., Negri E. (2015). European cancer mortality predictions for the year 2015: Does lung cancer have the highest death rate in EU women?. Ann. Oncol. Off. J. Eur. Soc. Med. Oncol..

[15] Noronha V., Ramaswamy A., Patil V.M., Joshi A., Chougule A., Kane S., Kumar R., Sahu A., Doshi V., Nayak L. (2016). ALK positive lung cancer: Clinical profile, practice and outcomes in a developing country. PloS ONE.

[16] Aberle D.R., Brown K. (2008). Lung cancer screening with CT. Clin. Chest Med..

[17] Journy N., Rehel J.L., Pointe H.D.L., Lee C., Brisse H., Chateil J.F., Caer-Lorho S., Laurier D., Bernier M.O. (2015). Are the studies on cancer risk from ct scans biased by indication? Elements of answer from a large-scale cohort study in France. Br. J. Cancer.

[18] Church T.R., Black W.C., Aberle D.R., Berg C.D., Clingan K.L., Duan F., Fagerstrom R.M., Gareen I.F., Gierada D.S., Jones G.C. (2013). Results of initial low-dose computed tomographic screening for lung cancer. N. Engl. J. Med..

[19] Asselin M.C., O’Connor J.P.B., Boellaard R., Thacker N.A., Jackson A. (2012). Quantifying heterogeneity in human tumours using MRI and PET. Eur. J. Cancer.

[20] Chicklore S., Goh V., Siddique M., Roy A., Marsden P.K., Cook G.J.R. (2013). Quantifying Tumour Heterogeneity in F-18-FDG PET/CT Imaging by Texture Analysis. Eur. J. Nucl. Med. Mol. Imaging.

[21] Ippolito D., Capraro C., Guerra L., De Ponti E., Messa C., Sironi S. (2013). Feasibility of perfusion CT technique integrated into conventional (18) FDG/PET-CT studies in lung cancer patients: Clinical staging and functional information in a single study. Eur. J. Nucl. Med. Mol. Imaging.

[22] Griffiths H. (2011). Magnetic induction tomography. Meas. Sci. Technol..

[23] Cheng B.Y. (2016). Development of a chemiluminescent immunoassay for cancer antigen 15-3. Labeled Immunoass. Clin. Med..

[24] Zhong L., Coe S.P., Stromberg A.J., Khattar N.H., Jett J.R., Hirschowitz E.A. (2006). Profiling tumor-associated antibodies for early detection of non-small cell lung cancer. J. Thorac. Oncol..

[25] Iizuka S., Taniguchi N., Makita A. (1984). Enzyme-linked immunosorbent assay for human manganese-containing superoxide dismutase and its content in lung cancer. J. Natl. Cancer Inst..

[26] Wang L. (2017). Early Diagnosis of Breast Cancer. Sensor.

[27] Eftekhari-Sis B., Aliabad M.A., Karimi F. (2016). Graphene oxide based nano-biosensor for the detection of deletion mutation in exon 19 of egfr gene, leading to lung cancer. Mater. Lett..

[28] Arya S.K., Bhansali S. (2011). Lung cancer and its early detection using biomarker-based biosensors. Chem. Rev..

[29] Xu X.W., Weng X.H., Wang C.L., Lin W.W., Liu A.L., Chen W., Xin X.H. (2016). Detection egfr exon 19 status of lung cancer patients by DNA electrochemical biosensor. Biosens. Bioelectron..

[30] Altintas Z., Tothill I. (2013). Biomarkers and biosensors for the early diagnosis of lung cancer. Sens. Actuators B Chem..

[31] Ghosal R., Kloer P., Lewis K.E. (2009). A review of novel biological tools used in screening for the early detection of lung cancer. Postgrad. Med. J..

[32] Gould M.K., Donington J., Lynch W.R., Mazzone P.J., Midthun D.E., Naidich D.P., Wiener R.S. (2013). Evaluation of individuals with pulmonary nodules: When is it lung cancer? Diagnosis and management of lung cancer: American College of Chest Physicians evidence based clinical practice guidelines. Chest J..

[33] Carlile P. (2015). Lung cancer screening: Where have we been? Where are we going?. J. Okla. State Med. Assoc..

[34] Latifi K., Dilling T.J., Feygelman V., Moros E.G., Stevens C.W., Montilla-Soler J.L., Zhang G.G. (2015). Impact of dose on lung ventilation change calculated from 4D-ct using deformable image registration in lung cancer patients treated with SBRT. J. Radiat. Oncol..

[35] Dajac J., Kamdar J., Moats A., Nguyen B. (2016). To screen or not to screen: Low dose computed tomography in comparison to chest radiography or usual care in reducing morbidity and mortality from lung cancer. Cureus.

[36] Rowan K.R., Kirkpatrick A.W., Liu D., Forkheim K.E., Mayo J.R., Nicolaou S. (2002). Traumatic pneumothorax detection with thoracic us: Correlation with chest radiography and CT--initial experience. Radiology.

[37] Karabulut N., Törü M., Gelebek V., Gülsün M., Ariyürek M.O. (2002). Comparison of low-dose and standard-dose helical CT in the evaluation of pulmonary nodules. Eur. Radiol..

[38] Kudo H. (2014). Image reconstruction methods in low-dose CT: Fundamentals of statistical image reconstruction, iterative image reconstruction, and compressed sensing. Med. Imaging Technol..

[39] Bach P.B., Jett J.R., Pastorino U., Tockman M.S., Swensen S.J., Begg C.B. (2007). Computed tomography screening and lung cancer outcomes. J. Am. Med. Assoc..

[40] Blanchon T., Br_echot J.-M., Grenier P.A., Ferretti G.R., Lemarié E., Milleron B., Chagué D., Laurent F., Martinet Y., Beigelman-Aubry C. (2007). Baseline results of the Depiscan study: A French randomized pilot trial of lung cancer screening comparing low dose CT scan (LDCT) and chest X-ray (CXR). Lung Cancer.

[41] Mcnittgray M.F. (2011). Reduced lung-cancer mortality with low-dose computed tomographic screening. N. Engl. J. Med..

[42] Saghir Z., Dirksen A., Ashraf H., Bach K.S., Brodersen J., Clementsen P.F., Døssing M., Hansen H., Kofoed K.F., Larsen K.R. (2012). CT screening for lung cancer brings forward early disease. The randomised Danish Lung Cancer Screening Trial: Status after five annual screening rounds with low-dose CT. Thorax.

[43] Pastorino U., Rossi M., Rosato V., Marchianò A., Sverzellati N., Morosi C., Fabbri A., Galeone C., Negri E., Sozzi G. (2012). Annual or biennial CT screening versus observation in heavy smokers: 5-year results of the MILD trial. Eur. J. Cancer Prev..

[44] Dwamena B.A., Sonnad S.S., Angobaldo J.O., Wahl R.L. (1999). Metastases from non-small cell lung cancer: Mediastinal staging in the 1990s--meta-analytic comparison of PET and CT. Radiology.

[45] Gould M.K., Kuschner W.G., Rydzak C.E., Maclean C.C., Demas A.N., Shigemitsu H., Chan J.K., Owens D.K. (2003). Test performance of positron emission tomography and computed tomography for mediastinal staging in patients with non-small-cell lung cancer: A meta-analysis. Ann. Intern. Med..

[46] Shim S.S., Lee K.S., Kim B.T., Chung M.J., Lee E.J., Han J., Choi J.Y., Kwon O.J., Shim Y.M., Kim S. (2005). Non-small cell lung cancer: Prospective comparison of integrated FDG PET/CT and CT alone for preoperative staging. Radiology.

[47] Al-Sarraf N., Gately K., Lucey J., Wilson L., Mcgovern E., Young V. (2008). Lymph node staging by means of positron emission tomography is less accurate in non-small cell lung cancer patients with enlarged lymph nodes: Analysis of 1145 lymph nodes. Lung Cancer.

[48] Ab G.D.C., Domínguez J.F., Bolton R.D., Pérez C.F., Martínez B.C., García-Esquinas M.G., Carreras Delgado J.L. (2017). PET-CT in presurgical lymph node staging in non-small cell lung cancer: The importance of false-negative and false-positive findings. Radiologia.

[49] Hellwig D., Ukena D., Paulsen F., Bamberg M., Kirsch C.M. (2001). Onko-PET der Deutschen Gesellschaft fur Nuklearmedizin. Meta-analysis of the efficacy of positron emission tomography with F-18-fluorodeoxyglucose in lung tumors. Basis for discussion of the German Consensus Conference on PET in Oncology 2000. Pneumologie.

[50] Ozeki Y., Abe Y., Kita H., Tamura K., Sakata I., Ishida J., Machida K. (2011). A case of primary lung cancer lesion demonstrated by F-18 FDG positron emission tomography/computed tomography (PET/CT) one year after the detection of metastatic brain tumor. Oncol. Lett..

[51] Yaturu S., Patel R.A. (2014). Metastases to the thyroid presenting as a metabolically inactive incidental thyroid nodule with stable size in 15 months. Case Rep. Endocrinol..

[52] Fischer B.M., Siegel B.A., Weber W.A., Von B.K., Beyer T., Kalemis A. (2016). PET/CT is a cost-effective tool against cancer: Synergy supersedes singularity. Eur. J. Nucl. Med. Mol. Imaging.

[53] Mac Manus M.P., Hicks R.J., Ball D.L., Kalff V., Matthews J.P., Salminen E., Khaw P., Wirth A., Rischin D., McKenzie A. (2001). F-18 fluorodeoxyglucose positron emission tomography staging in radical radiotherapy candidates with nonsmall cell lung carcinoma: Powerful correlation with survival and high impact on treatment. Cancer.

[54] Mac Manus M.P., Everitt S., Bayne M., Ball D., Plumridge N., Binns D., Herschtal A., Cruickshank D., Bressel M., Hicks R.J. (2013). The use of fused PET/CT images for patient selection and radical radiotherapy target volume definition in patients with non-small cell lung cancer: Results of a prospective study with mature survival data. Radiother. Oncol..

[55] Eschmann S.M., Friedel G., Paulsen F., Reimold M., Hehr T., Budach W., Langen H.J., Bares R. (2007). 18F-FDG PET for assessment of therapy response and preoperative re-evaluation after neoadjuvant radio-chemotherapy in stage III non-small cell lung cancer. Eur. J. Nucl. Med. Mol. Imaging.

[56] Lee W.K., Lau E.W., Chin K., Sedlaczek O., Steinke K. (2013). Modern diagnostic and therapeutic interventional radiology in lung cancer. J. Thorac. Dis..

[57] Zurek M., Bessaad A., Cieslar K., Crémillieux Y. (2010). Validation of simple and robust protocols for high-resolution lung proton MRI in mice. Magn. Reson. Med. Off. J. Soc. Magn. Reson. Med..

[58] Capaldi D., Sheikh K., Hoover D., Yaremko B., Palma D., Parraga G. (2016). Th-cd-202-09: Free-breathing proton MRI functional lung avoidance maps to guide radiation therapy. Med. Phys..

[59] Takahashi M., Togao O., Obara M., Cauteren M.V., Ohno Y., Doi S., Kuro-o M., Malloy C., Hsia C.C., Dimitrov I. (2010). Ultra-short echo time (UTE) MR imaging of the lung: Comparison between normal and emphysematous lungs in mutant mice. J. Magn. Reson. Imaging.

[60] Biederer J., Reuter M., Both M., Muhle C., Grimm J., Graessner J., Heller M. (2002). Analysis of artefacts and detail resolution of lung MRI with breath-hold t1-weighted gradient-echo and t2-weighted fast spin-echo sequences with respiratory triggering. Eur. Radiol..

[61] Burris N.S., Johnson K.M., Larson P.E.Z., Hope M.D., Nagle S.K., Behr S.C., Hope T.A. (2016). Detection of small pulmonary nodules with ultrashort echo time sequences in oncology patients by using a PET/MR system. Radiology.

[62] Fink C., Puderbach M., Biederer J., Fabel M., Dietrich O., Kauczor H.U., Reiser M.F., Schönberg S.O. (2007). Lung MRI at 1.5 and 3 tesla: Observer preference study and lesion contrast using five different pulse sequences. Investig. Radiol..

[63] Cieszanowski A., Anyszgrodzicka A., Szeszkowski W., Kaczynski B., Maj E., Gornicka B., Grodzicki M., Grudzinski I.P., Stadnik A., Krawczyk M. (2012). Characterization of focal liver lesions using quantitative techniques: Comparison of apparent diffusion coefficient values and T2 relaxation times. Eur. Radiol..

[64] Hughes D., Tiddens H., Wild J.M. (2009). Lung imaging in cystic fibrosis. Imaging Decis. MRI.

[65] Groth M., Henes F.O., Bannas P., Muellerleile K., Adam G., Regier M. (2011). Intraindividual comparison of contrast-enhanced MRI and unenhanced SSFP sequences of stenotic and non-stenotic pulmonary artery diameters. Rofo.

[66] Chong A.L., Chandra R.V., Chuah K.C., Roberts E.L., Stuckey S.L. (2016). Proton density MRI increases detection of cervical spinal cord multiple sclerosis lesions compared with T2-weighted fast spin-echo. AJNR Am. J. Neuroradiol..

[67] Mazzone P.J. (2008). Analysis of volatile organic compounds in the exhaled breath for the diagnosis of lung cancer. J. Thorac. Oncol..

[68] Nardi-Agmon I., Peled N. (2017). Exhaled breath analysis for the early detection of lung cancer: Recent developments and future prospects. Lung Cancer.

[69] Sakumura Y., Koyama Y., Tokutake H., Hida T., Sato K., Itoh T., Akamatsu T., Shin W. (2017). Diagnosis by volatile organic compounds in exhaled breath from lung cancer patients using support vector machine algorithm. Sensors.

[70] Lourenco C., Turner C. (2014). Breath analysis in disease diagnosis: Methodological considerations and applications. Metabolites.

[71] Phillips M., Gleeson K., Hughes J.M.B., Greenberg J., Cataneo R.N., Baker L., McVay W.P. (1999). Volatile organic compounds in breath as markers of lung cancer: A cross-sectional study. Lancet.

[72] Machado R.F., Laskowski D., Deffenderfer O., Burch T., Zheng S., Mazzone P.J., Mekhail T., Jennings C., Stoller J.K., Pyle J. (2005). Detection of lung cancer by sensor array analyses of exhaled breath. Am. J. Respir. Crit. Care Med..

[73] Mazzone P.J., Wang X.-F., Xu Y., Mekhail T., Beukemann M.C., Na J., Kemling J.W., Suslick K.S., Sasidhar M. (2012). Exhaled breath analysis with a colorimetric sensor array for the identification and characterization of lung cancer. J. Thorac. Oncol..

[74] Phillips M., Cataneo R.N., Cummin A.R., Gagliardi A.J., Gleeson K., Greenberg J., Maxfield R.A., Rom W.N. (2003). Detection of lung cancer with volatile markers in the breath. Chest J..

[75] Barash O., Peled N., Tisch U., Bunn P.A., Hirsch F.R., Haick H. (2012). Classification of lung cancer histology by gold nanoparticle sensors. Nanomedicine.

[76] Phillips M., Altorki N., Austin J.H., Cameron R.B., Cataneo R.N., Kloss R., Maxfield R.A., Munawar M.I., Pass H.I., Rashid A. (2008). Detection of lung cancer using weighted digital analysis of breath biomarkers. Clin. Chim. Acta.

[77] Phillips M., Altorki N., Austin J.H., Cameron R.B., Cataneo R.N., Greenberg J., Kloss R., Maxfield R.A., Munawar M.I., Pass H.I. (2007). Prediction of lung cancer using volatile biomarkers in breath. Cancer Biomark..

[78] Di Natale C., Macagnano A., Martinelli E., Paolesse R., D’Arcangelo G., Roscioni C., Finazzi-Agrò A., D’Amico A. (2003). Lung cancer identification by the analysis of breath by means of an array of non-selective gas sensors. Biosens. Bioelectron..

[79] Mazzone P.J., Hammel J., Dweik R., Na J., Czich C., Laskowski D., Mekhail T. (2007). Diagnosis of lung cancer by the analysis of exhaled breath with a colorimetric sensor array. Thorax.

[80] Itoh T., Miwa T., Tsuruta A., Akamatsu T., Izu N., Shin W., Park J., Hida T., Eda T., Setoguchi Y. (2016). Development of an Exhaled Breath Monitoring System with Semiconductive Gas Sensors, a Gas Condenser Unit, and Gas Chromatograph Columns. Sensors.

[81] Tricoles G., Farhat N.H. (1997). Microwave holography—Applications and techniques. Proc. IEEE.

[82] Han M., Cheng X., Xue Y. (2016). Comparison with reconstruction algorithms in magnetic induction tomography. Physiol. Meas..

[83] Hebel C.V., Rudolph S., Mester A., Huisman J.A., Kumbhar P., Vereecken H., van der Kruk J. (2014). Three-dimensional imaging of subsurface structural patterns using quantitative large-scale multiconfiguration electromagnetic induction data. Water Resour. Res..

[84] Ma L., Hunt A., Soleimani M. (2015). Experimental evaluation of conductive flow imaging using magnetic induction tomography. Int. J. Multiph. Flow.

[85] Zulkarnay Z., Abdul R.R., Badri M.M.S., Sazali Y., Nor A.N.M., Mohd M.S.Z., Muji S.Z.M., Rahiman M.H.F., Aman S.M.K.S. (2012). Advancements in transmitters and sensors for biological tissue imaging in magnetic induction tomography. Sensors.

[86] Binns R., Lyons A.R.A., Peyton A.J., Pritchard W.D.N. (2001). Imaging molten steel flow process. Meas. Sci. Technol..

[87] Alzeibak S., Saunders N.H. (1993). A feasibility study of in vivo electromagnetic imaging. Phys. Med. Biol..

[88] Karbeyaz B.U., Gencer N.G. (2003). Electrical conductivity imaging via contactless measurements: An experimental study. IEEE Trans. Med. Imaging.

[89] Riedel C.H., Keppelen M., Nani S., Merges R.D., Dössel O. (2004). Planar system for magnetic induction conductivity measurement using a sensor matrix. Physiol. Meas..

[90] Xu Z., Luo H., He W., He C., Song X., Zhang Z. (2003). A multi-channel magnetic induction tomography measurement system for human brain model imaging. Physiol. Meas..

[91] Ketchen M.B., Wolfgang K., Goubau M., Clarke J., Donaldson G.B. (1978). Superconducting thin-film gradiometer. J. Appl. Phys..

[92] Stolz R., Fritzsch L., Meyer H.G. (1999). LTS SQUID sensor with a new configuration. Supercond. Sci. Technol..

[93] Cantor R., Hall A., Matlachov A. (2006). Thin-film planar gradiometer with long baseline. J. Phys. Conf. Ser..

[94] Scharfetter H., Lackner H.K., Rosell J. (2001). Magnetic induction tomography: Hardware for multi-frequency measurements in biological tissues. Physiol. Meas..

[95] Rosell J., Casañas R., Scharfetter H. (2001). Sensitivity maps and system requirements for magnetic induction tomography using a planar gradiometer. Physiol. Meas..

[96] Scharfetter H., Rauchenzauner S., Merwa R., Biró O., Hollaus K. (2004). Planar gradiometer for magnetic induction tomography (MIT): Theoretical and experimental sensitivity maps for a low-contrast phantom. Physiol. Meas..

[97] Scharfetter H., Merwa R., Pilz K. (2005). A new type of gradiometer for the receiving circuit of magnetic induction tomography (MIT). Physiol. Meas..

[98] Merwa R., Hollaus K., Brunner P., Scharfetter H. (2006). Solution of the inverse problem of magnetic induction tomography (MIT). Physiol. Meas..

[99] Griffiths H., Holder D. (2005). Magnetic Induction Tomography. Electrical Impedance Tomography: Methods, History and Applications.

[100] Tumanski S. (2007). Induction Coil Sensors—A Review. Meas. Sci. Technol..

[101] Yu Z.Z., Peyton A.J., Xu L.A., Beck M.S. (1998). Electromagnetic inductance tomography (EMT): Sensor, electronics and image reconstruction for a system with a rotatable parallel excitation. IEE Proc. Sci. Meas. Technol..

[102] Mansor M.S.B., Zakaria Z., Balkhis I., Rahim R.A., Sahib M.F.A., Yunos Y.M., Sahlan S., Bunyamin S., Abas K.H., Ishak M.H.I. (2015). Magnetic induction tomography: A brief review. J. Teknol..

[103] Stawicki K., Gratkowski S., Komorowski M., Pietrusewicz T. (2009). A new transducer for magnetic induction tomography. IEEE Trans. Magn..

[104] Barba P.D., Mognaschi M.E., Palka R., Savini A. (2009). Optimization of the MIT field exciter by a multiobjective design. IEEE Trans. Magn..

[105] Watson S., Wee H.C., Griffiths H., Williams R.J. (2011). A Highly Phase-Stable Differential Detector Amplifier for Magnetic Induction Tomography. Physiol. Meas..

[106] Igney C.H., Watson S., Williamson S.J., Griffiths H., Dössel O. (2005). Design and performance of a planar-array MIT system with normal sensor alignment. Physiol. Meas..

[107] Eichardt E., Igney C.H., Kahlert J., Hamsch M., Vauhkonen M., Haueisen J. (2009). Sensitivity comparisons of cylindrical and hemi-spherical coil setups for magnetic induction tomography. IFMBE Proc. World Conf..

[108] Gursoy D., Scharfetter H. (2009). Optimum receiver array design for magnetic induction tomography. IEEE Trans. Biomed. Eng..

[109] Scharfetter H., Issa I., Gürsoy D. (2010). Tracking of object movements for artefact suppression in Magnetic Induction Tomography (MIT). J. Phys. Conf. Ser..

[110] Marmugi L., Renzoni F. (2016). Optical magnetic induction tomography of the heart. Sci. Rep..

[111] Hu G., Cressman E., He B. (2011). Magnetoacoustic imaging of human liver tumor with magnetic induction. Appl. Phys. Lett..

[112] Żywica A.R. (2016). Magnetoacoustic tomography with magnetic induction for biological tissue imaging: Numerical modelling and simulations. Arch. Electr. Eng..

[113] Watson S., Williams R.J., Griffiths H., Gough W., Morris A. (2003). Magnetic induction tomography: Phase versus vector-voltmeter measurement techniques. Physiol. Meas..

[114] Vauhkonen M., Hamsch M., Igney C.H. (2008). A measurement system and image reconstruction in magnetic induction tomography. Physiol. Meas..

[115] Gabriel S., Lau R.W., Gabriel C. (1996). The dielectric properties of biological tissues: II. Measurements in the frequency range 10 Hz to 20 GHz. Phys. Med. Biol..

[116] Gabriel S., Lau R.W., Gabriel C. (1996). The dielectric properties of biological tissues: III. Parametric models for the dielectric spectrum of tissues. Phys. Med. Biol..

[117] Patz R., Watson S., Ktistis C., Hamsch M., Peyton A.J. (2010). Performance of a FPGA-based Direct Digitising Signal Measurement module for MIT. J. Phys. Conf. Ser..

[118] Wei H.Y., Soleimani M. (2012). Theoretical and experimental evaluation of rotational magnetic induction tomography. IEEE Trans. Instrum. Meas..

[119] Scharfetter H., Kostinger A., Issa S. (2008). Hardware for Quasi-Single-Shot Multifrequency Magnetic Induction Tomography (MIT): The Graz Mk2 System. Physiol. Meas..

[120] Wang L., Al-Jumaily A.M. (2017). Imaging of lung structure using holographic electromagnetic induction. IEEE Access..

[121] Villalobos P., Wistuba I.I. (2017). Lung cancer biomarkers. Hematol/Oncol. Clin. N. Am..

[122] Jia J.W., Li K.L., Wu J.X., Guo S.L. (2013). Clinical significance of annexin ii expression in human non-small cell lung cancer. Tumour Biol..

[123] Uribarri M., Hormaeche I., Zalacain R., Lopezvivanco G., Martinez A., Nagore D., Ruiz-Argüello M.B. (2014). A new biomarker panel in bronchoalveolar lavage for an improved lung cancer diagnosis. J. Thorac. Oncol..

[124] Dong Y., Zheng X., Yang Z., Sun M., Zhang G., An X., Pan L., Zhang S. (2016). Serum carcinoembryonic antigen, neuron-specific enolase as biomarkers for diagnosis of nonsmall cell lung cancer. J. Cancer Res. Ther..

[125] Gube M., Taeger D., Weber D.G., Pesch B., Brand P., Johnen G., Müller-Lux A., Gross I.M., Wiethege T., Weber A. (2011). Performance of biomarkers smrp, ca125, and cyfra 21-1 as potential tumor markers for malignant mesothelioma and lung cancer in a cohort of workers formerly exposed to asbestos. Arch. Toxicol..

[126] Huang Z., Jiang Z., Zhao C., Han W., Lin L., Liu A., Weng S., Lin X. (2017). Simple and effective label-free electrochemical immunoassay for carbohydrate antigen 19-9 based on polythionine-au composites as enhanced sensing signals for detecting different clinical samples. Int. J. Nanomed..

[127] So H.J., Hong S.I., Lee J.K., Chang Y.H., Kang S.J., Hong Y.J. (2014). Comparison of the serum fibrin-fibrinogen degradation products with cytokeratin 19 fragment as biomarkers in patients with lung cancer. Biomed. Rep..

[128] Li B., Lin H., Fan J., Lan J., Zhong Y., Yang Y., Li H., Wang Z. (2013). Cd59 is overexpressed in human lung cancer and regulates apoptosis of human lung cancer cells. Int. J. Oncol..

[129] Ding H., Liu J., Xue R., Zhao P., Qin Y., Zheng F., Sun X. (2014). Transthyretin as a potential biomarker for the differential diagnosis between lung cancer and lung infection. Biomed. Rep..

[130] Potprommanee L., Ma H.T., Shank L., Juan Y.H., Liao W.Y., Chen S.T., Yu C.J. (2015). Gm2-activator protein: A new biomarker for lung cancer. J. Thorac. Oncol..

[131] Wang B., He Y.J., Tian Y.X., Yang R.N., Zhu Y.R., Qiu H. (2014). Clinical utility of haptoglobin in combination with cea, nse and cyfra21-1 for diagnosis of lung cancer. Asian Pac. J. Cancer Prev..

[132] Kormelink T.G., Powe D.G., Kuijpers S.A., Abudukelimu A., Fens M.H.A.M., Pieters E.H.E., der Ven W.W.K., Habashy H.O., Ellis I.O., Blokhuis B.R. (2014). Immunoglobulin free light chains are biomarkers of poor prognosis in basal-like breast cancer and are potential targets in tumor-associated inflammation. Oncotarget.

[133] Zhou Y., Chen W.Z., Peng A.F., Tong W.L., Liu J.M., Liu Z.L. (2017). Neuron-specific enolase, histopathological types, and age as risk factors for bone metastases in lung cancer. Tumour Biol..

[134] Martin Mateo M.C., Bustamante B.J., Font A.I. (1979). Serum copper, ceruloplasmin, lactic-dehydrogenase and alpha 2-globulin in lung cancer. Biomedicine.

[135] Chee J., Naran A., Misso N.L., Thompson P.J., Bhoola K.D. (2008). Expression of tissue and plasma kallikreins and kinin b1 and b2 receptors in lung cancer. Biol. Chem..

[136] Winther B., Reubsaet J.L. (2015). Determination of the small cell lung cancer associated biomarker pro-gastrin-releasing peptide (progrp) using lc-ms. J. Sep. Sci..

[137] Wang T., Liang Y., Thakur A., Zhang S., Liu F., Khan H., Shi P., Wang N., Chen M., Ren H. (2017). Expression and clinicopathological significance of s100 calcium binding protein a2 in lung cancer patients of chinese han ethnicity. Clin. Chim. Acta.

[138] Wang T., Zhang L., Tian P., Tian S. (2017). Identification of differentially-expressed genes between early-stage adenocarcinoma and squamous cell carcinoma lung cancer using meta-analysis methods. Oncol. Lett..

[139] Loftus T.J., Thomson A.J., Kannan K.B., Alamo I.G., Ramos H.N., Whitley E.E., Efron P.A., Mohr A.M. (2017). Effects of trauma, hemorrhagic shock, and chronic stress on lung vascular endothelial growth factor. J. Surg. Res..

[140] Foa P., Fornier M., Miceli R., Seregni E., Santambrogio L., Nosotti M., Cataldo I., Sala M., Caldiera S., Bombardieri E. (1999). Tumour markers cea, nse, scc, tpa and cyfra 21.1 in resectable non-small cell lung cancer. Anticancer Res..

[141] Liu J., Zhu H., Jiang H., Zhang H., Wu D., Hu X., Zhang H. (2015). Tumor m2 pyruvate kinase in diagnosis of nonsmall cell lung cancer: A meta-analysis based on Chinese population. J. Cancer Res. Ther..

[142] Indovina P., Marcelli E., Pentimalli F., Tanganelli P., Tarro G., Giordano A. (2013). Mass spectrometry-based proteomics: The road to lung cancer biomarker discovery. Mass Spectrom. Rev..

[143] Ye F., Shi M.Y., Zhao S. (2010). Noncompetitive immunoassay for carcinoembryonic antigen in human serum by microchip electrophoresis for cancer diagnosis. Clin. Chim. Acta.

[144] Schneider J., Philipp MVelcovsky H.G., Morr H., Katz N. (2003). Pro-gastrin-releasing peptide (progrp), neuron specific enolase (nse), carcinoembryonic antigen (cea) and cytokeratin 19-fragments (cyfra 21-1) in patients with lung cancer in comparison to other lung diseases. Anticancer Res..

[145] Ho J.A., Chang H.C., Shih N.Y., Wu L.C., Chang Y.F., Chen C.C., Chou C. (2010). Diagnostic detection of human lung cancer-associated antigen using a gold nanoparticle-based electrochemical immunosensor. Anal. Chem..

[146] Patz E.F., Campa M.J., Gottlin E.B., Kusmartseva I., Guan X.R., Nd H.J. (2007). Panel of serum biomarkers for the diagnosis of lung cancer. J. Clin. Oncol..

[147] Bennett W.P., Hussain S.P., Vahakangas K.H., Khan M.A., Shields P.G., Harris C.C. (1999). Molecular epidemiology of human cancer risk: Gene–environment interactions and p53 mutation spectrum in human lung cancer. J. Pathol..

[148] Ono A., Takahashi T., Mori K., Akamatsu H., Shukuya T., Taira T., Kenmotsu H., Naito T., Murakami H., Nakajima T. (2013). Prognostic impact of serum cyfra 21-1 in patients with advanced lung adenocarcinoma: A retrospective study. BMC Cancer.

[149] Liu L., Liu B., Zhu L.L., Li Y. (2013). Cyfra21-1 as a serum tumor marker for follow-up patients with squamous cell lung carcinoma and oropharynx squamous cell carcinoma. Biomark. Med..

[150] Zhao H., Shi X., Liu J., Chen Z., Wang G. (2014). Serum cyfra21-1 as a biomarker in patients with nonsmall cell lung cancer. J. Cancer Res. Ther..

[151] Zhang G., Xiaobo M.A., Liyi H.U., Zang J., Zhang G., Qinglei X.U. (2013). The application significance of serum cyfra21-1 change in therapeutic efficacy monitoring of advanced stage NSCLC. Lab. Med..

[152] Zereu M., Vinholes J.J., Zettler C.G. (2003). P53 and bcl-2 protein expression and its relationship with prognosis in small-cell lung cancer. Clin. Lung Cancer.

[153] Kim D.H., Nelson H.H., Wiencke J.K., Zheng S., Christiani D.C., Wain J.C., Mark E.J., Kelsey K.T. (2001). P16(ink4a) and histology-specific methylation of cpg islands by exposure to tobacco smoke in non-small cell lung cancer. Cancer Res..

[154] Kondo K., Takahashi Y., Hirose Y., Nagao T., Tsuyuguchi M., Hashimoto M., Ochiai A., Monden Y., Tangoku A. (2006). The reduced expression and aberrant methylation of p16(ink4a) in chromate workers with lung cancer. Lung Cancer.

[155] Belinsky S.A., Klinge D.M., Liechty K.C., March T.H., Kang T., Gilliland F.D., Sotnic N., Adamova G., Rusinova G., Telnov V. (2004). Plutonium targets the p16 gene for inactivation by promoter hypermethylation in human lung adenocarcinoma. Carcinogenesis.

[156] Cooper G.M. (1982). Cellular transforming genes. Science.

[157] Downward J. (2003). Targeting RAS signalling pathways in cancer therapy. Nat. Rev. Cancer.

[158] Kovalchuk O., Naumnik W., Serwicka A., Chyczewska E., Niklinski J., Chyczewski L. (2001). K-ras codon 12 mutations may be detected in serum of patients suffering from adeno- and large cell lung carcinoma. A preliminary report. Folia Histochem. Cytobiol..

[159] Iii H.H., Cawthon R., He X., Chanock S., Lan Q. (2009). Genetic variation in telomere maintenance genes, telomere length, and lung cancer susceptibility. Lung Cancer.

[160] Hu Z., Yang Z., Tian T., Liang J., Jin G., Shen H. (2009). Association between microrna polymorphisms, expressions, lung cancer development and prognosis. Biomed. Pharmacother..

[161] Schmitt M.J., Margue C., Behrmann I., Kreis S. (2013). Mirna-29: A microrna family with tumor-suppressing and immune-modulating properties. Curr. Mol. Med..

[162] Dacic S. (2012). Molecular Prognostic Markers of Lung Cancer. Molecular Pathology of Lung Cancer.

[163] Wu J.J., Yang T., Li X., Yang Q.Y., Liu R., Huang J.K., Li Y.Q., Yang C.F., Jiang Y.G. (2013). Alteration of serum mir-206 and mir-133b is associated with lung carcinogenesis induced by 4-(methylnitrosamino)-1-(3-pyridyl)-1-butanone. Toxicol. Appl. Pharmacol..

[164] Yanaihara N., Caplen N., Bowman E., Seike M., Kumamoto K., Yi M., Stephens R.M., Okamoto A., Yokota J., Tanaka T. (2006). Unique microrna molecular profiles in lung cancer diagnosis and prognosis. Cancer Cell.

[165] Miao L.J., Huang S.F., Sun Z.T., Gao Z.Y., Zhang R.X., Liu Y., Wang J. (2013). Mir-449c targets C-myc and inhibits NSCLC cell progression. FEBS Lett..

[166] Sezginturk M.K. (2011). A new impedimetric biosensor utilizing vegf receptor-1 (flt-1): Early diagnosis of vascular endothelial growth factor in breast cancer. Biosens. Bioelectron..

[167] Nonaka Y., Abe K., Ikebukuro K. (2012). Electrochemical detection of vascular endothelial growth factor with aptamer sandwich. Electrochemistry.

[168] Cho H., Yeh E.C., Sinha R., Laurence T.A., Bearinger J.P., Lee L.P. (2012). Single-step nanoplasmonic vegf(165) aptasensor for early cancer diagnosis. ACS Nano.

[169] Remy-Martin F., El Osta M., Lucchi G., Zeggari R., Leblois T., Bellon S., Ducoroy P., Boireau W. (2012). Surface plasmon resonance imaging in arrays coupled with mass spectrometry (supra-ms): Proof of concept of on-chip characterization of a potential breast cancer marker in human plasma. Anal. Bioanal. Chem..

[170] Altintas Z., Uludag Y., Gurbuz Y., Tothill I.E. (2011). Surface plasmon resonance based immunosensor for the detection of the cancer biomarker carcinoembryonic antigen. Talanta.

[171] Noah N.M., Mwilu S.K., Sadik O.A., Fatah A.A., Arcilesi R.D. (2011). Immunosensors for quantifying cyclooxygenase 2 pain biomarkers. Clin. Chim. Acta.

[172] Ladd J., Lu H., Taylor A.D., Goodell V., Disis M.L., Jiang S. (2009). Direct detection of carcinoembryonic antigen autoantibodies in clinical human serum samples using a surface plasmon resonance sensor. Colloids Surf. B.

[173] Zhou Y., Wang Z., Yue W., Tang K., Ruan W., Zhang Q., Liu L. (2009). Label-Free detection of p53 antibody using a microcantilever biosensor with piezoresistive readout. IEEE Sens..

[174] Wang X., Zhang X., He P., Fang Y. (2011). Sensitive detection of p53 tumor suppressor gene using an enzyme-based solid-state electrochemiluminescence sensing platform. Biosens. Bioelectron..

[175] Wang Y., Zhu X., Wu M., Xia N., Wang J., Zhou F. (2009). Simultaneous and label-free determination of wild-type and mutant p53 at a single surface plasmon resonance chip preimmobilized with consensus DNA and monoclonal antibody. Anal. Chem..

[176] Ilyas A., Asghar W., Allen P.B., Duhon H., Ellington A.D., Iqbal S.M. (2012). Electrical detection of cancer biomarker using aptamers with nanogap break-junctions. Nanotechnology.

[177] Chung J.W., Bernhardt R., Pyun J.C. (2006). Additive assay of cancer marker ca 19-9 by SPR biosensor. Sens. Actuators B.

[178] Ladd J., Taylor A.D., Piliarik M., Homola J., Jiang S. (2009). Label-free detection of cancer biomarker candidates using surface plasmon resonance imaging. Anal. Bioanal. Chem..

[179] Yokotani T., Koizumi T., Taniguchi R., Nakagawa T., Isobe T., Yoshimura M., Tsubota N., Hasegawa K., Ohsawa N., Baba S. (1997). Expression of α and β genes of human chorionic gonadotropin in lung cancer. Int. J. Cancer.

[180] Piliarik M., Bockova M., Homola J. (2010). Surface plasmon resonance biosensor for parallelized detection of protein biomarkers in diluted blood plasma. Biosens. Bioelectron..

[181] Carrascosa L.G., Calle A., Lechuga L.M. (2009). Label-free detection of DNA mutations by SPR: Application to the early detection of inherited breast cancer. Anal. Bioanal. Chem..

[182] Wang H., Wang X., Wang J., Fu W., Yao C. (2016). A SPR biosensor based on signal amplification using antibody-QD conjugates for quantitative determination of multiple tumor markers. Sci. Rep..

[183] Cheng S., Hideshima S., Kuroiwa S., Nakanishi T., Osaka T. (2015). Label-free detection of tumor markers using field effect transistor (FET)-based biosensors for lung cancer diagnosis. Sens. Actuators B Chem..

[184] Donzella V., Crea F. (2011). Optical biosensors to analyze novel biomarkers in oncology. J. Biophotonics.

[185] Uludag Y., Tothill I.E., Chem A. (2012). Cancer biomarker detection in serum samples using surface plasmon resonance and quartz crystal microbalance sensors with nanoparticle signal amplification. Anal. Chem..

[186] Wang B., Liu J.T., Luo J.P., Wang M.X., Shu-Xue Q.U., Cai X.X. (2013). A three-channel high-precision optical detecting system for lung cancer marker cyfra21-1. J. Optoelectron. Laser.

[187] Ribaut C., Loyez M., Larrieu J.C., Chevineau S., Lambert P., Remmelink M., Wattiez R., Christophe Caucheteur C. (2016). Cancer biomarker sensing using packaged plasmonic optical fiber gratings: Towards in vivo diagnosis. Biosens. Bioelectron..

[188] Liu L.S., Wu C., Zhang S. (2017). Ultrasensitive detection of DNA and ramos cell using in situ selective crystallization based quartz crystal microbalance. Anal. Chem..

[189] Heydari S., Haghayegh G.H. (2014). Application of nanoparticles in quartz crystal microbalance biosensors. J. Sens. Technol..

[190] Della V.B., Iannaccone M., Funari R., Pica C.M., Altucci C., Capparelli R., Roperto S., Velotta R. (2017). Effective antibodies immobilization and functionalized nanoparticles in a quartz-crystal microbalance-based immunosensor for the detection of parathion. PLoS ONE.

[191] Sun W., Song W., Guo X., Wang Z. (2017). Ultrasensitive detection of nucleic acids and proteins using quartz crystal microbalance and surface plasmon resonance sensors based on target-triggering multiple signal amplification strategy. Anal. Chim. Acta.

[192] Chen M., Hou C., Huo D., Yang M., Fa H. (2015). A highly sensitive electrochemical DNA biosensor for rapid detection of cyfra21-1, a marker of non-small cell lung cancer. Anal. Methods.

[193] Konvalina G., Haick H. (2014). Sensors for breath testing: From nanomaterials to comprehensive disease detection. Acc. Chem. Res..

[194] Liu S., Su W., Li Z., Ding X. (2015). Electrochemical detection of lung cancer specific micrornas using 3d DNA origami nanostructures. Biosens. Bioelectron..

[195] Altintas Z., Kallempudi S.S., Sezerman U., Gurbuz Y. (2012). A novel magnetic particle-modified electrochemical sensor for immunosensor applications. Sens. Actuators B Chem..

[196] Amouzadeh T.M., Shamsipur M., Farzin L. (2015). A high sensitive electrochemical aptasensor for the determination of VEGF (165) in serum of lung cancer patient. Biosens. Bioelectron..

[197] Zamay G.S., Zamay T.N., Kolovskii V.A., Shabanov A.V., Glazyrin Y.E., Veprintsev D.V., Krat A.V., Zamay S.S., Kolovskaya O.S., Gargaun A. (2016). Electrochemical aptasensor for lung cancer-related protein detection in crude blood plasma samples. Sci. Rep..

