# Correction: Screening and Biosensor-Based Approaches for Lung Cancer Detection. *Sensors* 2017, *17*, 2420

**DOI:** 10.3390/s19204393

**Published:** 2019-10-11

**Authors:** Lulu Wang

**Affiliations:** 1 School of Instrument Science and Opto-electronics Engineering, Hefei University of Technology, Hefei 230009, China; luluwang2015@hfut.edu.cn; 2 Institute of Biomedical Technologies, Auckland University of Technology, Auckland 1142, New Zealand

The authors wish to make the following corrections to this paper [1]:

In the Introduction section of the paper [1], up to 96.4% was mistakenly used in the sentences “To solve this limitation, LDCT was applied for lung imaging and it reduced 20% of lung cancer mortality [18]. However, LDCT continues to have a high false positive rate (up to 96.4%) [19].” So, the correct sentence is given below:

“To solve this limitation, LDCT was applied for lung imaging and it reduced 20% of lung cancer mortality [18,19].”

In the References section of the paper [1], the References 19 and 41 were mistakenly used, so the correct reference is given below:

19. National Lung Screening Trial Research Team. Reduced lung-cancer mortality with low-dose computed tomographic screening. *N. Engl. J. Med.* **2011**, *365*, 395–409.

41. Kaneko, M. Peripheral lung cancer: Screening and detection with low-dose spiral CT versus radiography. *Radiology* **1996**, *201*, 789–802.
